# Testicular Interposition Flap for Repair of Perineal Urinary Fistulae: A Novel Surgical Technique

**DOI:** 10.1155/2015/836454

**Published:** 2015-09-21

**Authors:** Valary T. Raup, Jairam R. Eswara, Stephen D. Marshall, Steven B. Brandes

**Affiliations:** ^1^Division of Urology, Department of Surgery, Brigham and Women's Hospital, Harvard Medical School, Boston, MA 02115, USA; ^2^Division of Urology, Department of Surgery, Washington University School of Medicine, St. Louis, MO 63110, USA

## Abstract

Rectourinary fistulae and urinary-cutaneous fistulae are a rare yet devastating complication. Current options for tissue interposition include rectus, gracilis, or gluteal muscle, omentum, or intestine for use in coloanal pull-through procedures. In elderly patients, testicular interposition flaps may be an excellent tissue option to use when vitalized tissue is necessary to supplement fistula repair. Elderly patients frequently have increased spermatic cord length, potentially offering a longer flap reach than use of a muscle flap. Additionally, mobilizing one of the testicles and developing it through the external inguinal ring may be a less morbid and less costly procedure than harvesting and tunneling a muscle flap. Longer follow-up and further studies are needed to determine the outcomes of this novel technique.

## 1. Introduction

Rectourinary fistulae and urinary-cutaneous fistulae are a rare yet devastating complication. The most common causes include iatrogenic injury from abdominal or pelvic surgery, trauma, pelvic radiation, or a complication of pelvic inflammatory diseases, infections, or malignancies [1, 2]. Excision of the fistula tract with primary anastomosis and advancement of adjacent tissue can be utilized to repair smaller fistulae, but larger fistulae may require a greater volume of vitalized tissue for interposition. Options for tissue interposition include rectus, gracilis, or gluteal muscle, omentum, or intestine for use in coloanal pull-through procedures [3, 4]. The viability of these surgical options varies due to location and size of the fistulae, local anatomy, and availability of tissue. Here, we report a novel surgical approach of testicular mobilization and interposition to repair two cases of perineal fistulae.

## 2. Case Report #1

**Figure 1:**
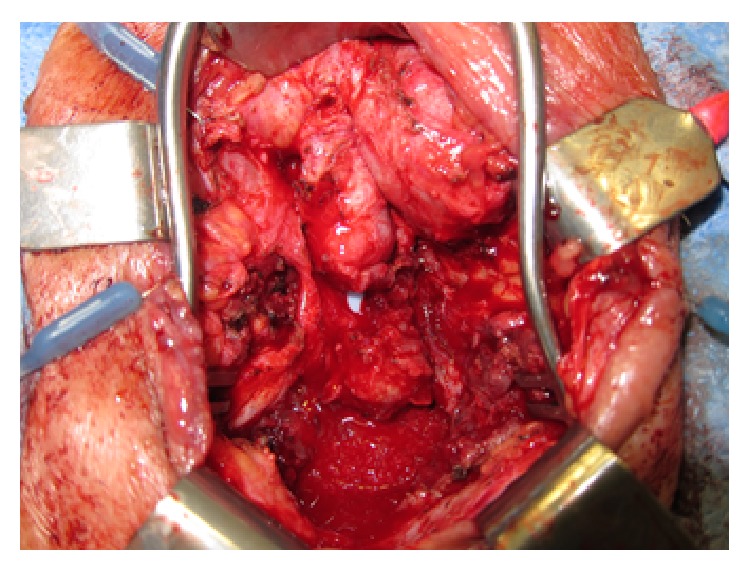
Urethral fistula with visible Foley catheter.

A 72-year-old Caucasian male presented with constant perineal urinary leakage. He was found to have a 2 cm urethrocutaneous fistula caused by an infected, eroded mesh urethral sling placed two years previously to treat urinary incontinence. This fistula was verified with cystoscopy and he was scheduled for operative fistula repair. He was given preoperative vancomycin and Unasyn and a Foley catheter was placed under direct visualization with cystoscopic guidance. A midline perineal incision was made in order to identify the urethra, and tissue planes were found to be quite distorted by fibrosis and scarring. The sling was removed, leaving a substantial area of dead space (Figure 1).

**Figure 2:**
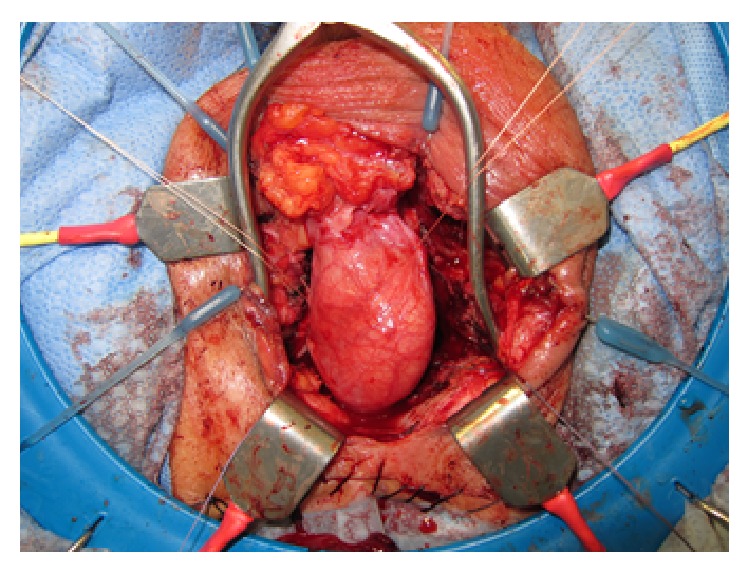
Testicle mobilized to external ring.

The urethra was mobilized both proximally and distally and then anastomosed end-to-end with multiple interrupted 2-0 Vicryl sutures. The right testicle was then mobilized to the level of the external ring and interposed to both cover the urethral repair and fill the defect (Figure 2).

**Figure 3:**
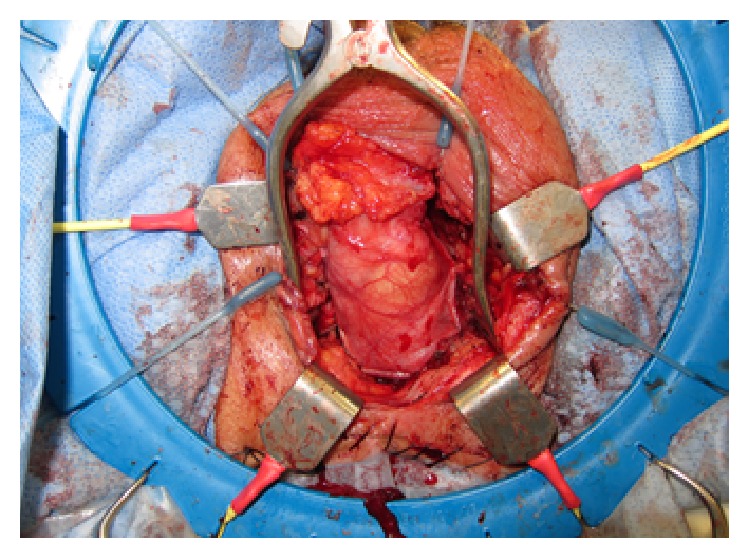
Testicular interposition flap tacked down with multiple 2-0 Vicryl sutures.

The testicle was tacked into place with multiple 2-0 Vicryl sutures and the resulting closure of the defect was deemed to be favorable (Figure 3).

Flexible cystoscopy was performed to confirm closure of the fistula, and the wound was then closed in three layers with 2-0 and 3-0 Vicryl sutures. Compressive fluff dressings were applied and a Foley catheter was placed and hung to gravity drainage.

The patient did not experience any symptoms postoperatively and was discharged after 2 days. He was sent home on 21 days of antibiotics and his Foley catheter was removed after 4 weeks. Fistula closure was verified by cystoscopy at 4 weeks, 6 months, and 1 year postoperatively. All incisions healed without complication, and the patient still denied testicular pain, urinary incontinence, and dysuria at 18-month follow-up.

## 3. Case Report #2

An 87-year-old Caucasian male presented with urethral stump recurrence of T2N3 transitional cell carcinoma of the bladder, status postinduction chemotherapy, and radical cystoprostatectomy/ileal conduit diversion, who presented with pelvic lymphadenectomy three years earlier. This recurrence was found on routine surveillance imaging and verified with cystoscopy. He was taken to the operating room shortly thereafter for resection of the tumor via an open perineal approach. Given the extensive nature of the urethral stump recurrence, an iatrogenic rectal injury of 8 mm × 4 mm was incurred during urethrectomy. This defect was repaired primarily with interrupted 0 Vicryl sutures, but the rectal tissue was deemed to be quite thin. A proctoscope was placed in the rectum and closure of the defect was assessed by filling the wound with water and insufflating the rectum with air. The defect was found to be successfully approximated. In order to fill the defect space, we mobilized the right tunica vaginalis and cremasteric fibers of the testicle up to the external ring and tacked these tissues onto the rectum in multiple areas. A significant dead space remained, which we felt needed to be filled with vascularized tissue. A gracilis flap was considered, but the left testicle offered adequate bulk with effortless tension-free reach. The left testicle was mobilized to the level of the external ring and fit perfectly in the dead space underneath the pubic rami. We tacked the testis into place with 2-0 Vicryl sutures and were quite pleased with the overall result. Colorectal surgery was consulted to confirm the integrity of the rectal repair. The scrotum was closed in three layers with 2-0/3-0 Vicryl and 3-0 chromic sutures. A 15 Fr Blake drain was placed and brought out through a separate incision, and the perineum was closed in three layers with 2-0 and 3-0 Vicryl sutures. The urethra was closed with interrupted sutures, Foley catheter was replaced, compressive dressings were placed, and the patient was subsequently transferred to the recovery room in stable condition.

Postoperatively, the patient complained of diarrhea, which completely resolved after one week. Upon discharge, he denied rectal or urinary incontinence, dysuria, blood per rectum, testicular pain, or pain with bowel movements. He was sent home with 21 days of antibiotics. The Blake drain remained in place for 2 weeks, and the Foley catheter remained in place until the 6-week follow-up visit. All incisions healed without complications. Fistula closure was verified with cystoscopy at 6 weeks, 6 months, and 1 year postoperatively. At 18-month follow-up, the patient was still symptom-free.

## 4. Discussion

The use of tissue flaps is an important aspect of urinary fistula repair. Often, the tissues around the fistula are scarred and devitalized necessitating the interposition of healthy, vascularized tissue to maintain adequate blood flow to the area and to protect the repair [5]. Gracilis and rectus muscle are typically used to fill dead space in perineal fistula repairs due to their length, ease of harvest, and consistent vascular anatomy. The use of a gracilis muscle flap does not typically result in noticeable thigh weakness or decreased range of motion due to the redundancy of similar muscles in the thigh. However, symptoms of thigh numbness and harvest site wound infection have been described [6, 7]. Additionally, gracilis muscle repairs have a variable success rate of 73–90% reported in the literature [8–10]. Patients often require a second gracilis flap fistula repair to achieve complete healing, and success rates of 100% have been reported in single-institution series in which both gracilis muscles are used in the initial repair [11]. While the use of both gracilis muscles provides a higher rate of successful repair, it increases the morbidity of the procedure and leaves few options if repeat repair is necessary.

The testicle is a highly mobile, well-vascularized organ with enough bulk to fill substantial dead space during perineal fistula repair. In elderly patients who are poor candidates for gracilis flap repair or have already utilized their gracilis muscles in prior repairs, testicular interposition flaps may be a reasonable alternative. Both of our patients experienced complete fistula closure without appreciable postoperative complications, and outcomes in 1.5–2 years were outstanding. These results suggest that this procedure has excellent potential and merits additional attention.

As with any major surgical procedure, patients must have acceptable overall health in order to undergo surgery and recover successfully. Mobilizing one of the testicles and developing it through the external inguinal ring is faster and less morbid than harvesting and tunneling a muscle flap. Additionally, the tunica vaginalis surrounding the testicle provides an additional layer for closure. Large thigh wounds are replaced with a small scrotal incision, and the procedure is less expensive due to decreased time in the operating room. Also, it is imperative that the gracilis muscle flap fills the perineal defect without being placed under tension. If the neurovascular bundle is under tension, the flap may become ischemic and the fistula will likely recur. Elderly patients frequently have increased spermatic cord length, potentially offering a longer flap reach than with use of a muscle flap. The testicle may also offer greater bulk than the gracilis flap in elderly patients who have experienced muscle wasting due to inactivity.

Potential complications of this procedure include testicular pain and the possibility of testicular cancer in younger patients. It is possible that subjecting the testicle to a warmer environment may increase the risk of infertility and testicular cancer development [12, 13]. Patients with cryptorchidism who are treated after the age of 12 are 2–6 times more likely to develop testicular cancer [14]. It is unclear whether this technique mimics cryptorchidism since, in patients undergoing fistula repair, the testicles descended normally during development and were subject to an abdominopelvic environment after reaching adulthood. Longer follow-up and analysis of additional cases are required to determine the success and potential complications of this novel procedure.

## 5. Conclusion

Definitive management of perineal urinary fistulae should be decided based on patient-specific factors such as life expectancy, premorbid urinary and bowel function, continence, local anatomy, and the technical feasibility of a repair. Testicular interposition flaps may be an excellent tissue option to use when vascularized tissue is necessary to supplement fistula repair. Longer follow-up and further studies are needed to determine the outcomes of this novel technique.
